# Cannabidiol causes endothelium-dependent vasorelaxation of human mesenteric arteries via CB_1_ activation

**DOI:** 10.1093/cvr/cvv179

**Published:** 2015-06-19

**Authors:** Christopher P. Stanley, William H. Hind, Cristina Tufarelli, Saoirse E. O'Sullivan

**Affiliations:** School of Medicine, University of Nottingham, Royal Derby Hospital, DerbyDE22 3DT, UK

**Keywords:** Vasorelaxation, Human, Cannabidiol, Cannabinoid, Endothelium

## Abstract

**Aims:**

The protective effects of cannabidiol (CBD) have been widely shown in preclinical models and have translated into medicines for the treatment of multiple sclerosis and epilepsy. However, the direct vascular effects of CBD in humans are unknown.

**Methods and results:**

Using wire myography, the vascular effects of CBD were assessed in human mesenteric arteries, and the mechanisms of action probed pharmacologically. CBD-induced intracellular signalling was characterized using human aortic endothelial cells (HAECs). CBD caused acute, non-recoverable vasorelaxation of human mesenteric arteries with an *R*_max_ of ∼40%. This was inhibited by cannabinoid receptor 1 (CB_1_) receptor antagonists, desensitization of transient receptor potential channels using capsaicin, removal of the endothelium, and inhibition of potassium efflux. There was no role for cannabinoid receptor-2 (CB_2_) receptor, peroxisome proliferator activated receptor (PPAR)γ, the novel endothelial cannabinoid receptor (CB_e_), or cyclooxygenase. CBD-induced vasorelaxation was blunted in males, and in patients with type 2 diabetes or hypercholesterolemia. In HAECs, CBD significantly reduced phosphorylated JNK, NFκB, p70s6 K and STAT5, and significantly increased phosphorylated CREB, ERK1/2, and Akt levels. CBD also increased phosphorylated eNOS (ser1177), which was correlated with increased levels of ERK1/2 and Akt levels. CB_1_ receptor antagonism prevented the increase in eNOS phosphorylation.

**Conclusion:**

This study shows, for the first time, that CBD causes vasorelaxation of human mesenteric arteries via activation of CB_1_ and TRP channels, and is endothelium- and nitric oxide-dependent.

## 1. Introduction

Numerous studies have shown that endogenous, synthetic, and plant-derived cannabinoids cause vasorelaxation of a range of animal and human arterial beds.^1,2^ The extent of cannabinoid-induced vasorelaxation and the mechanisms involved often differs between the cannabinoid compound studied, the arterial bed used, and the species employed. These mechanisms include activation of cannabinoid receptor one (CB_1_), cannabinoid receptor two (CB_2_), transient receptor potential vanilloid one (TRPV1), peroxisome proliferator activated receptor gamma (PPARγ), and an as yet unidentified endothelial-bound cannabinoid receptor (CB_e_).^1,2^ Vasorelaxant mediators implicated in cannabinoid-induced vasorelaxation include nitric oxide production, prostaglandin production, metabolite production, and ion channel modulation, some of which have been shown to be coupled to receptor activation.^1,2^

Cannabidiol (CBD) is a naturally occurring molecule found in the plant *Cannabis sativa*. Unlike the related molecule Δ^9^-tetrahydrocannabinol (THC), it does not activate CB_1_ receptors in the brain, and is devoid of the psychotropic actions of THC. Indeed, CBD may antagonize the psychoses associated with cannabis abuse.^3^ Other receptor sites implicated in the actions of CBD include the orphan G-protein-coupled receptor GPR55, the putative endothelial cannabinoid receptor (CB_e_), the transient receptor potential vanilloid 1 (TRPV1) receptor, α1-adrenoceptors, µ opioid receptors and 5-HT_1A_ receptors,^4,5^ A CBD/THC combination (1 : 1 ratio, Sativex/Nabiximols) is currently licensed internationally in more than 20 countries for the treatment of spasticity in multiple sclerosis, and an as yet unlicensed CBD alone product (Epidiolex) has entered an expanded access programme in children with intractable epilepsies. CBD has also received orphan designation status in treating newborn children with neonatal hypoxic-ischaemic encephalopathy.

In addition to the licensed indications, preclinical evidence suggests CBD has therapeutic potential in diseases associated with inflammation, oxidative stress, gastrointestinal disturbances, neurodegeneration, cancer, diabetes, and nociception.^6–10^ In the cardiovascular system, CBD treatment *in vivo* reduces endothelial and cardiac dysfunction in cardiomyopathy associated with diabetes.^11,12^ CBD also reduces vascular inflammation associated with endotoxic shock,^13^ has a protective role in diabetic retinopathy,^14^ and is cardioprotective after coronary artery ligation.^15^ Furthermore, CBD reduces infarct size and increases cerebral blood flow in a mouse model of stroke when delivered either pre- or post-ischaemia through activation of 5-HT_1A_ receptors.^16–19^

Unlike other cannabinoids, the direct vascular effects of CBD have not been fully investigated in either animal or human studies.^1^ Jarai *et al.* ^20^ showed that CBD (10 μmol/L) had no effect on vascular tone in the perfused mesenteric arterial bed of mice. However, Offertaler *et al*.^21^ reported that CBD caused a concentration-dependent near maximal vasorelaxation of isolated rat mesenteric arteries, but no mechanisms of action were probed. In the rat isolated aorta, we showed that CBD causes a time-dependant vasorelaxant response that was inhibited by antagonism of the PPARγ receptor and inhibition of superoxide dismutase.^22^

In light of the increasing evidence that CBD has beneficial effects on the cardiovascular system, and since the vascular effects of CBD remain to be characterized in human vasculature, the aim of the present study was to establish the acute vascular effects of CBD in human arteries and to underpin the pharmacology behind any potential response.

## 2. Methods

Ethical approval was granted by the Derbyshire Research Ethics Committee and Derbyshire Hospitals Trust Research and Development to take mesenteric tissue from patients (27 males, 10 females) undergoing colorectal surgery. Informed consent was gained in accordance with the Declaration of Helsinki. Mesenteric arteries have been extensively used to characterize the pharmacological effects of cannabinoids.^1^ Excised mesenteric tissue was placed in physiological saline solution (PSS) and transported back to the lab. Arteries (701 ± 42 µm diameter, mean ± SEM) were dissected from mesenteric tissue, cleaned of any adherent fatty and connective tissue and cut into 2 mm segments. Artery segments were either used fresh or after overnight storage in PSS at 4°C. Overnight storage had no significant effect on the contractile or relaxation responses of mesenteric arteries (see Supplementary material online, *Figure S1*). Artery segments were mounted on tungsten wires on a Mulvany-Halpern myograph (Danish Myo Technology, Denmark) at 37°C in PSS solution and gassed with 5% CO_2_ in O_2_. Tension was measured using isometric force displacement transducers and recorded using Chart 5 Pro (ADinstruments, Oxfordshire, UK). Using normalization software, arteries were set to an internal diameter producing 90 mmHg pressure. To establish artery viability, the ability of arteries to contract to high potassium PSS (KPSS) (composition, mmol/L: NaCl 0, KCl 124, CaCl_2_ · 2H_2_O 2.5, MgSO_4_ · 7H_2_O 1.17, NaHCO_3_ 25, KH_2_PO_4_ 1.18, C_10_H_16_N_2_O_8_ 0.027, C_6_H_12_O_6_ 5.5 all dissolved in triple distilled water) or to contract to U46619 (>5 mM), and to relax to 10 μmol/L bradykinin (>70% relaxation) was measured.

### 2.1 Experimental protocol

Viable arteries were contracted using a combination of U46619 (50–250 nmol/L) and Endothelin-1 (1–3 nmol/L). Once a stable contraction >5 mN was achieved, cumulative concentration–response curves were constructed to CBD. CBD was added in 5-min intervals with measurements taken in the final minute of each concentration addition and expressed as percentage relaxation of pre-imposed tone. Responses were compared with ethanol-treated vehicle controls carried out in adjacent arterial segments from the same patient. To characterize mechanisms underpinning CBD-induced vasorelaxation, all interventions were compared with a CBD control–response carried out in adjacent arteries from the same patient. In some experiments, the endothelium was removed by abrasion using a human hair. A role for the involvement of nitric oxide was investigated using NG-nitro-l-arginine methyl ester (l-NAME, 300 μmol/L, present throughout). A role for cyclooxygenase (COX) was assessed using indomethacin (10 μmol/L, present throughout). A potential role for potassium channel hyperpolarization was investigated by carrying out concentration–response curves to CBD in arteries contracted using KPSS to inhibit potassium efflux. Potential cannabinoid receptor involvement in CBD-induced vasorelaxation was assessed with CB_1_ antagonist (AM251, 100 nmol/L or LY320135 1 μmol/L), CB_2_ receptor antagonist AM630 (100 nmol/L), or proposed endothelial cannabinoid receptor (CB_e_, O1918, 10 μmol/L). Desensitization of sensory nerves was achieved via incubation (1 h) with capsaicin (10 μmol/L) followed by three washouts in PSS. In experiments to establish the potential location of the CB_1_ receptor, the effects of AM251 in endothelial-denuded arteries were compared with arteries that were endothelial denuded only, arteries treated with AM251 only and CBD control arteries. In each of these protocols, there was no significant difference in the level of contraction immediately before the CBD concentration response curve.

### 2.2 Cell culture

Human aortic endothelial cells (PromoCell, Germany, passage 4) were grown in PromoCell Endothelial Cell Growth medium to confluence on 6-well plates and treated for 10 min with increasing concentrations of CBD, after which time the medium was removed and the cells collected in cell lysis buffer (RIPA buffer, SigmaAldrich) with phosphatase and protease inhibitors (Roche). Some experiments were performed in the presence of AM251 or capsazepine. The protein concentration of the cell lysate was measured using a BCA assay (BCA-1KT, SigmaAldrich). The levels of phosphorylated ERK/MAP kinase 1/2 (Thr185/Tyr187), Akt (Ser473), STAT3 (Ser727), JNK (Thr183/Tyr185), p70 S6 kinase (Thr412), NFkB (Ser536), STAT5A/B (Tyr694/699), CREB (Ser133), and p38 (Thr180/Tyr182) were measured in cell lysates using the Luminex® xMAP^®^ technology using a commercially available panel (Milliplex™, 48-680MAG, Merck Millipore), and normalized to total protein content. eNOS phosphorylation was measured using a PathScan Phospho-eNOS (ser1177) sandwich ELISA according to the manufacturer's instructions (Cell Signaling Technology, USA), and was normalized to total protein content.

### 2.3 Reverse transcription-polymerase chain reaction

The presence of target sites of action was investigated at the mRNA level using reverse transcription followed by polymerase chain reaction (RT-PCR) under control conditions, and in the presence of a high glucose (25 mM) or high insulin (500 nM) medium for 96 h. Human astrocytes (HAs) were used as a positive control known to express all the target sites of action of interest.^23^ Total RNA was extracted from HAs and HAECs using Allprep DNA/RNA kit with on column DNaseI treatment (Qiagen, Germany). Reverse transcription with and without reverse transcriptase was performed in 30 µl final volume using 3 µg of total RNA and random primers with the High Capacity cDNA Reverse Transcription Kit (Life Technologies, UK) according to the manufacturer's instructions. PCRs were carried out in a final volume of 25 µl with Zymotaq (ZymoResearch, USA) using 2 µl of reverse transcription product as the template. Primer pairs used to amplify 128 bp of the control house-keeping gene Hypoxanthine-guanine PhosphoRibosylTransferase (HPRT) were from ref. 24; those for 99 bp PPARα and 87 bp PPARγ were from ref. 25; those for 303 bp CB1R and 365 bp CB2R were from ref. 26; those for 511 bp TRPV1 were from ref. 27; and finally the 380 bp calcitonin gene-related peptide (CGRP) receptor (CGRPR) cDNA fragment was amplified using the primers reported in ref. 28. After 5 min at 95°C, PCRs were performed for 40 cycles except those for CB2R that was carried out for 50 cycles. The cycles included 30 s at 95°C, 30 s at the annealing temperature that was optimal for each primer pair (56°C for CB1R and CB2R; 60°C for all others) and a final extension step of 30 s at 72°C. Amplification products were separated by gel electrophoresis through ethidium bromide stained 2% agarose (CB1R, CB2R, TRPV1, CGRPR) and 3% NuSieve 3:1 (PPARα, PPARγ and HPRT) and visualized using a Biorad Chemidoc.

### 2.4 Statistical analysis

Graphs represent mean percentage relaxations, with error bars representing the standard error of the mean (SEM) fit to non-linear Regression (Curve Fit) (Prism Version 6; GraphPad Software, CA, USA). *n* represents the number of arteries from patients. Comparisons between intervention and control artery segments from the same patient were made using *R*_max_ (the calculated maximal response to CBD) and EC_50_ (potency of CBD) compared by Student's *t*-test. In experiments to assess the location of the CB_1_ receptor, comparisons were made between artery segments from the same patient using one way analysis of variance (ANOVA) with Dunnetts post-hoc analysis. Significance was determined as *P* < 0.05.

### 2.5 Chemicals

All salts, l-NAME, indomethacin and bradykinin were supplied by Sigma Chemical Co. (Poole, UK). AM251, LY 320135, AM630, and capsaicin were purchased from Tocris (Bristol, UK). CBD was a kind gift from GW Pharmaceuticals (Wiltshire, UK). l-NAME and indomethacin were dissolved in PSS solution. CBD, bradykinin, and capsaicin were all dissolved in ethanol at 10 mM with further dilutions made in distilled water. AM251, LY320135, and AM630 were dissolved in DMSO at 10 mmol/L with further dilutions made in distilled water.

## 3. Results

Thirty-four patients (24 males and 10 females) were recruited for this study. Twenty-seven had cancer and 7 had inflammatory bowel disorder. A summary of patient characteristics, medical history, and medications is presented in *Table 1*.

**Table 1:** Patient characteristics, diagnosis, and medications

Characteristic	Range	Mean ± SEM
Ethnicity	34 UK white	
Male	24	
Female	10	
Age	36–82	65 ± 2.1
Weight (kg)	52–126	76 ± 3
BMI (kg/m^2^)	17.5–36.4	27.1 ± 0.7
Vessel size (µm)	346–1372	701 ± 42
Bradykinin response (% relaxation)	70–109	85 ± 1.4
Smoking habits		
Non-smokers	28	
0–10 CPD	3	
10–20 CPD	3	
Drinking habits		
< 10 units p/w	23	
10–20 units p/w	7	
> 20 units p/w	4	
Operation		
Right hemicolectomy	10	
Left hemicolectomy	7	
Sigmoid colectomy	5	
Anterior resection	10	
Abdominoperineal resection	1	
Total colectomy	1	
Reason for surgery		
Cancer	27	
Inflammatory bowel disorder	7	
Dukes Staging		
Dukes A	10	
Dukes B	9	
Dukes C	8	
Dukes D	0	
Systolic blood pressure (mm/Hg)	110–188	143 ± 3
Diastolic blood pressure (mm/Hg)	62–101	82 ± 1
Diabetic	10	
Heart disease	9	
Heart failure	0	
Hypercholesterolemia	15	
Hypertensive	16	
α-1-adrenoceptor antagonist	3	
ACE inhibitors	7	
AT1 receptor antagonists	2	
Beta-blockers	6	
Calcium-channel blocker	3	
Digoxin	2	
Diuretics	3	
GTN	3	
Hypoglycaemic medication	6	
Nsaid medication	14	
Statin	14	
Thiazolidinedione	1	

**Figure 1:**
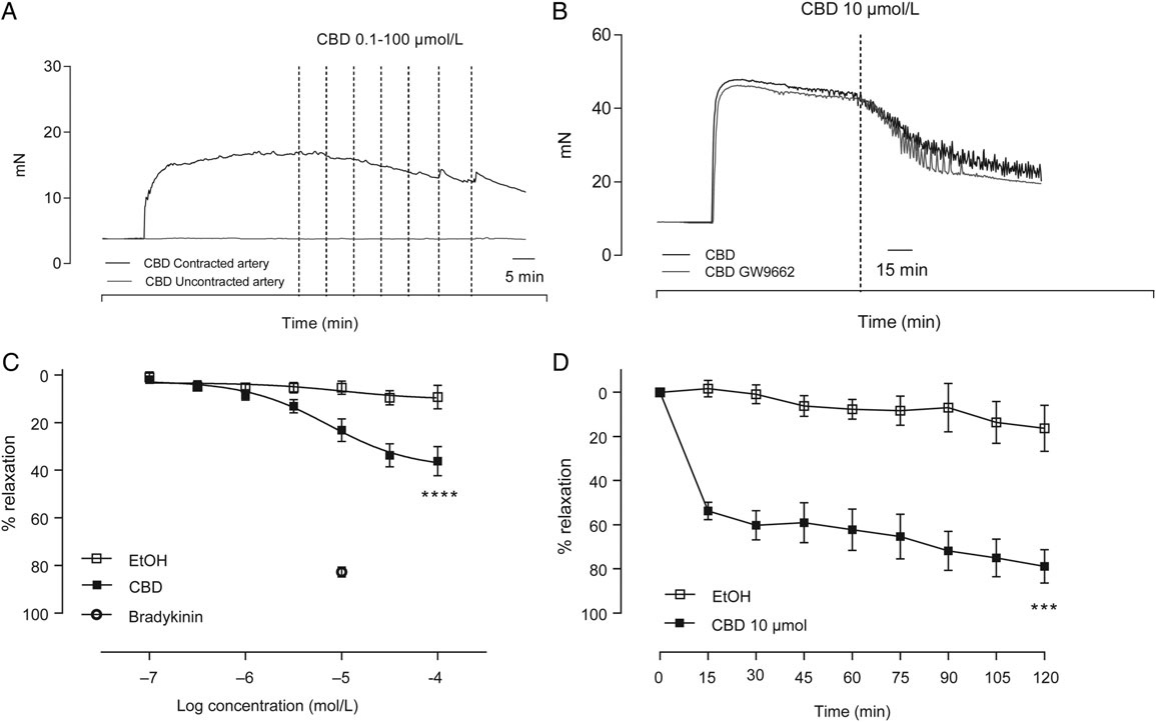
CBD relaxes human mesenteric arteries. Typical trace data showing the acute (*A*) and time-dependent (*B*) vasorelaxant effects of CBD (also in the presence of the PPARgamma antagonist GW9662) in the human mesenteric artery. (*C*) Mean (± SEM, *n* = 12) concentration-response curves to CBD compared with vehicle controls carried out in adjacent segments of mesenteric artery from the same patient. The vasorelaxant response to 10 µmol/L bradykinin in the same patients is shown for comparison. (*D*) Mean time-dependent vasorelaxant response to a single concentration of CBD (10 µmol/L) compared with vehicle controls carried out in adjacent segments of mesenteric artery (*n* = 6). *R*_max_ and EC_50_ values were compared by paired Students *t-*test, *P < 0.05, ****P < 0.0001.

CBD caused vasorelaxation of pre-constricted human mesenteric arteries with an *R*_max_ of around 40% vasorelaxation (*R*_max_ *P* < 0.0001 compared with vehicle control, *n* = 12, *Figure 1A* and *C*, *Table 2*). For comparison, the vasorelaxant response to 10 µmol/L bradykinin (83 ± 3 (mean ± SEM) % relaxation) in the same patients is represented in *Figure 1C*. When added to un-contracted arteries, CBD had no effect on baseline tone (*n* = 6, representative raw trace shown in *Figure 1A*). In time-dependent experiments, a single concentration of 10 µmol/L CBD caused an initial vasorelaxation of 57 ± 4% relaxation at 15 min, developing to 78 ± 7% at 120 min (*P* < 0.001, *n* = 6, *Figure 1D*).

**Table 2:** The maximal vasorelaxant responses and potency of CBD in human mesenteric arteries

		Vehicle	CBD	*n*
	*R*_max_	10.2 ± 3.5	39.2 ± 4.0 ****	
	EC_50_	−4.98 ± 0.87	−5.14 ± 0.21	12
		Control CBD	Intervention	*n*
Minus endothelium	*R*_max_	51.6 ± 2.8	44.6 ± 3.8	
	EC_50_	−5.84 ± 0.18	−5.21 ± 0.18 ****	8
L-NAME	*R*_max_	51.4 ± 4.9	39.1 ± 6.6	
	EC_50_	−5.39 ± 0.26	−5.24 ± 0.35	6
Indomethacin	*R*_max_	50.4 ± 4.0	55.2 ± 4.6	
	EC_50_	−5.82 ± 0.26	−5.26 ± 0.20	6
KPSS contracted	*R*_max_	49.7 ± 5.8	8.9 ± 2.4 ***	
	EC_50_	−5.45 ± 0.30	−5.59 ± 0.73	5
AM251	*R*_max_	53.9 ± 3.7	24.2 ± 4.9 ***	
	EC_50_	−5.57 ± 0.19	−5.53 ± 0.49	9
LY320135	*R*_max_	45.0 ± 3.5	30.2 ± 5.4 *	
	EC_50_	−5.83 ± 0.24	−5.88 ± 0.54	6
AM630	*R*_max_	58.7 ± 3.9	59.5 ± 5.5	
	EC_50_	−5.56 ± 0.17	−5.48 ± 0.23	8
Capsaicin pre-treatment	*R*_max_	47.7 ± 2.4	21.3 ± 3.9 ****	
	EC_50_	−5.92 ± 0.15	−5.85 ± 0.39	7
O-1918	*R*_max_	51.8 ± 2.8	43.8 ± 3.9	
	EC_50_	−5.68 ± 0.16	−5.61 ± 0.26	7

Sigmoidal concentration-response curves to CBD were fitted using Prism and *R*_max_ and EC_50_ values were compared by Student's t test (with Welch's correction for groups with unequal standard deviations).

**Figure 2:**
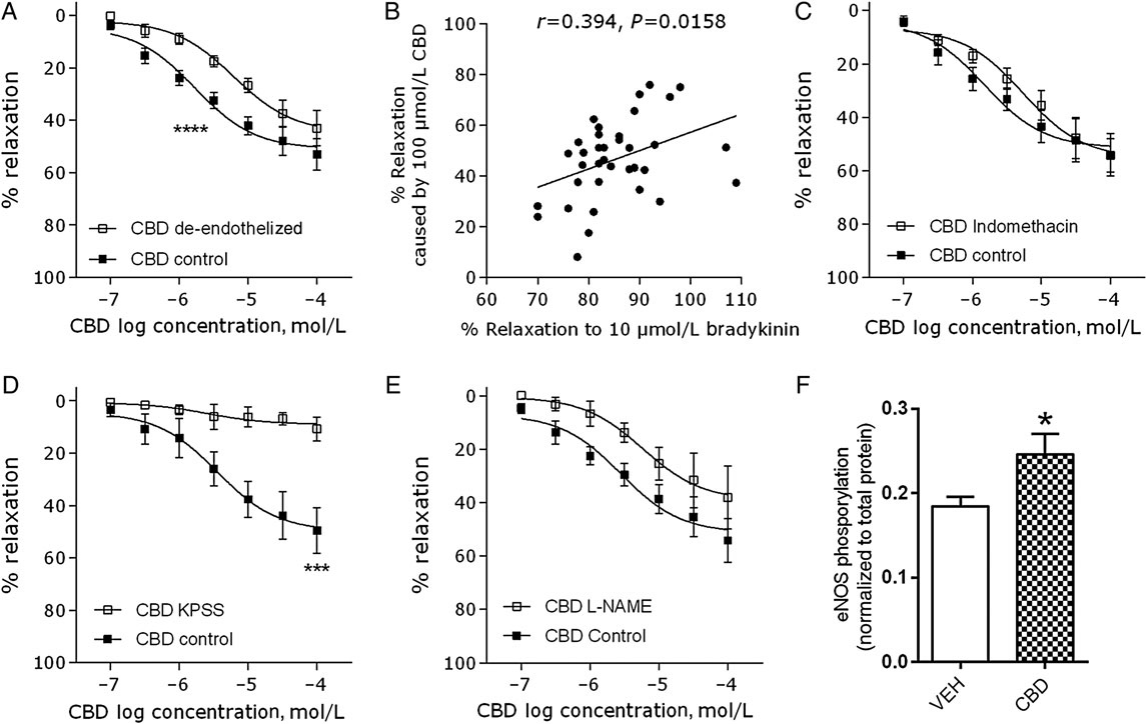
Mechanisms of CBD-induced relaxation of human mesenteric arteries. Mean (± SEM) CBD-induced vasorelaxation of human mesenteric arteries after removal of the endothelium (*n* = 8, *A*), in arteries incubated with l-NAME (300 µmol/L, *n* = 6, *B*), in the presence of the non-selective COX inhibitor indomethacin (10 µmol/L, *n* = 6, *D*) or in arteries contracted using a high potassium (KPSS) Krebs (*n* = 5, *E*). (*C*) Maximal responses to CBD correlated with the vasorelaxant response to the endothelium-dependent vasorelaxant bradykinin. (*F*) In cultured human aortic endothelial cells, CBD (10 µmol/L, 10 min) increased eNOS phosphorylation at ser1177 (*n* = 9). Control responses to CBD and interventions were carried out in adjacent segments of mesenteric artery from the same patient. *R*_max_ and EC_50_ values were compared by paired Students *t-*test, *P < 0.05, **P < 0.01, ***P < 0.001, ****P < 0.0001.

Removal of the endothelium significantly reduced the potency (EC_50_) of CBD (*P* < 0.0001, *Figure 2A*, *Table 2*). The maximum vasorelaxation to CBD also correlated positively with the endothelium-dependent bradykinin response in patients (*r* = 0.394, *P* = 0.0158, *Figure 2B*). Inhibition of COX activity using indomethacin had no effect on the CBD-induced vasorelaxation (*n* = 6, *Figure 2C*). In arteries contracted using high potassium physiological salt solution (KPSS), CBD-induced vasorelaxation was significantly inhibited (*R*_max_ *P* < 0.001, *n* = 5 *Figure 2D*). Although incubation with l-NAME did not significantly affect the concentration–response curve to CBD (*Figure 2B*, *Table 2*), a trend for a reduction in the vasorelaxant effect of CBD was seen. Therefore, in cultured endothelial cells, we tested whether CBD affects eNOS activation and found that CBD (10 µmol/L, 10 min) significantly increased eNOS phosphorylation at ser1177 (*P* < 0.05, *n* = 9, *Figure 2F*). Neither endothelium-denudation, l-NAME, or KPSS contraction affected control vasorelaxant responses (see Supplementary material online, *Figure S2*).

**Figure 3:**
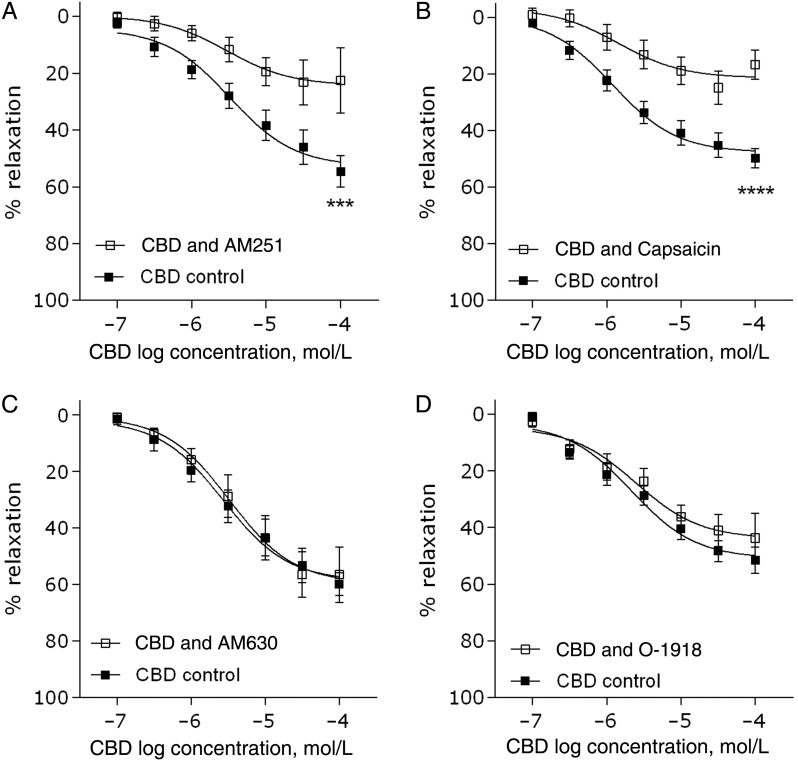
Target sites of action for CBD-induced relaxation of human mesenteric arteries. CBD-induced vasorelaxation of human mesenteric arteries after 10 min incubation (pre-contraction) with the CB_1_ antagonist AM251 (100 nmol/L, *n* = 9, *A*), the CB_2_ antagonist AM630 (100 nmol/L, *n* = 8, *C*), the proposed endothelial receptor (CB_e_) antagonist O-1918 (10 µmol/L, *n* = 7, *D*), or after desensitization of sensory nerves by 1 h pre-treatment with the TRPV1 agonist capsaicin (10 μmol/L, *n* = 7, *B*). Control responses to CBD and interventions were carried out in adjacent segments of mesenteric artery from the same patient. *R*_max_ and EC_50_ values were compared by paired Students *t-*test ,*P < 0.05, **P < 0.01, ***P < 0.001, ****P < 0.0001.

Antagonism of the CB_1_ receptor using AM251 (100 nmol/L) significantly inhibited CBD-induced vasorelaxation (*R*_max_ *P* < 0.001, *n* = 9, *Figure 3A*, *Table 2*). To confirm this result, a second, structurally different antagonist LY320135 was used, which also significantly reduced the maximal response to CBD (CBD *R*_max_ 45 ± 3.5; CBD&LY *R*_max_ 30 ± 5.4, *P* < 0.05, *Table 2*). Antagonism of the CB_2_ receptor using AM630 (100 nmol/L) had no effect on CBD-induced vasorelaxation (*n* = 8, *Figure 3C*). Desensitization of TRP channels using capsaicin (10 μmol/L) reduced CBD-induced vasorelaxation (*P* < 0.0001, *n* = 7, *Figure 3B*). Antagonism of the proposed CB_e_ receptor using O-1918 (10 µmol/L, *n* = 7, *Figure 3D*) had no effect on the CBD-induced vasorelaxation. In the presence of the PPARγ antagonist GW9662, neither the immediate nor the time-dependent vasorelaxation was inhibited (*n* = 5, representative raw trace shown in *Figure 1B*). Neither AM251, LY320135, or capsaicin pre-treatment affected control vasorelaxant responses (see Supplementary material online, *Figure S2*).

**Figure 4:**
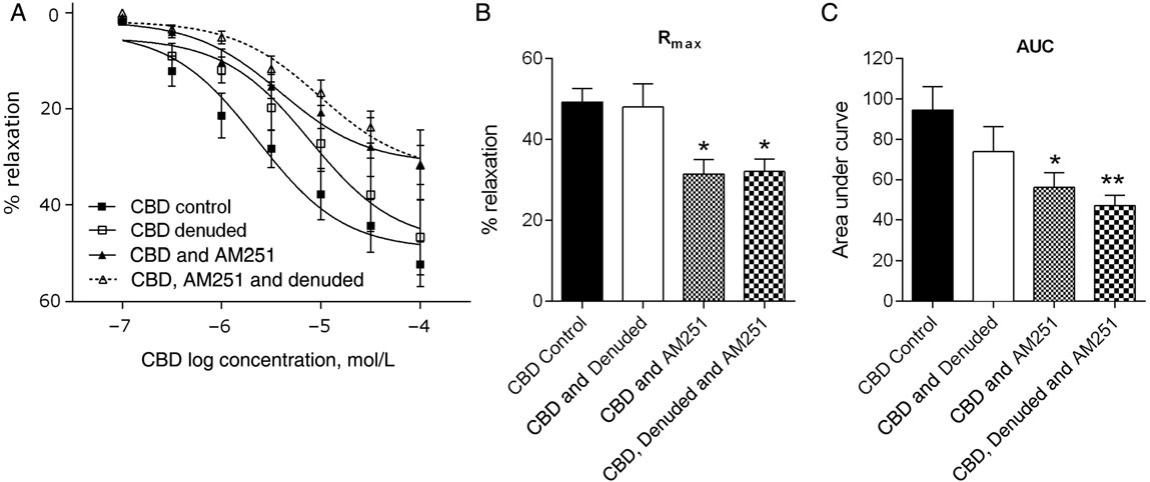
Location of the CB_1_ receptor. Mean CBD-induced vasorelaxation in control arteries, endothelial denuded arteries, in arteries incubated with the CB_1_ antagonist AM251 or in arteries that are endothelial denuded and incubated with AM251 (*A*) and the corresponding *R*_max_ (*B*) and AUC (*C*) values within each patient (*n* = 6). Control responses to CBD and the three interventions were carried out in adjacent segments of mesenteric artery from the same patient. Data were compared using one way analysis of variance (ANOVA) with Dunnett's *post hoc* analysis comparing against the CBD control data. *P < 0.05, **P < 0.01.

In experiments to determine the location of the CB_1_ receptor, AM251, and endothelial denudation were compared in combination and individually against control CBD responses, obtained from adjacent segments of artery from the same patients (*n* = 6, *Figure 4A*). AM251 alone, and AM251 plus denudation, resulted in a significant reduction in the maximal response (*R*_max_) to CBD to similar extent (*P* < 0.05, *Figure 4C*). However, when looking at the entire concentration response curve to CBD (AUC values), the combination of AM251 and endothelial denudation had a more significant (*P* < 0.01) reduction than AM251 alone (*P* < 0.05, *Figure 4B*).

Across the 37 patients tested, considerable variability of control responses to CBD was observed among patients (the maximal response to CBD ranged from 2 to 75% relaxation), so *post hoc* analysis was carried out to establish any relationships between CBD responses and patient characteristics (see Supplementary material online, *Table S1* and *Figures 3* and *4*). CBD responses were slightly reduced in males compared with females (*P* = 0.0166), but were not affected by age, BMI, or smoking status. Looking at concurrent diseases, CBD responses were reduced in patients with type-2 diabetes (*P* < 0.0001), hypercholesterolemia (*P* = 0.0320), but not different in patients with cancer, heart disease, or hypertension (Supplementary material online, *Figure S4*). CBD responses were reduced in those taking statins (*P* = 0.0042), hypoglycaemic medication (*P* < 0.0001) and beta-blockers (*P* = 0.0094), but not those taking ACE inhibitors or NSAIDs (Supplementary material online, *Figure S4*).

**Figure 5:**
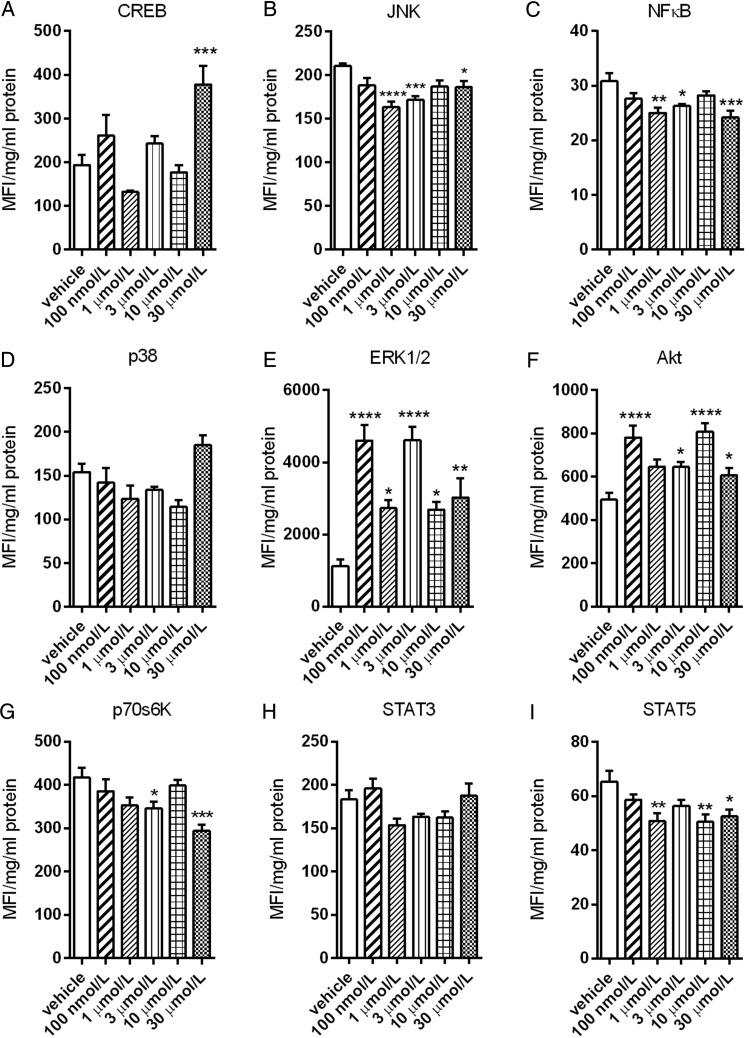
Signal transduction by CBD in human endothelial cells. Levels of phosphorylated CREB (*A*), JNK (*B*), NFκB (*C*), p38 (*D*), ERK/MAP kinase 1/2 (*E*), Akt (*F*), p70 S6 kinase (*G*), STAT3 (*H*), and STAT5A/B (*I*) were measured in human aortic endothelial cell lysates after 10 min treatment with increasing concentrations of CBD using the Luminex^®^ xMAP^®^ technology and normalized to total protein content. MFI, median fluorescent intensity. Data are presented as mean ± SEM (*n* = 6) and were analysed by ANOVA with Dunnett's *post-hoc* analysis against the vehicle control response. *P < 0.05, **P < 0.01, ***P < 0.001, ****P < 0.0001.

**Figure 6:**
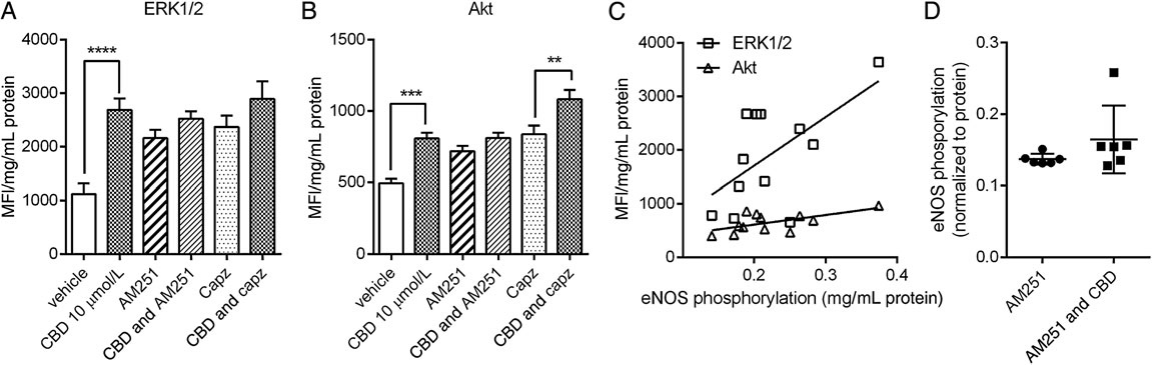
Signal transduction by CBD in human endothelial cells. Levels of phosphorylated ERK/MAP kinase 1/2 (*A*) and Akt (*B*) measured in human aortic endothelial cell lysates after 10 min treatment with CBD in the presence of the CB_1_ antagonist AM251 (100 nM) or the TRPV1 antagonist capzasepine (1 μM). (*C*) Correlation of levels of phosphorylated ERK1/2 and Akt with levels of phosphorylated eNOS in human aortic endothelial cell lysates after 10 min treatment with CBD. MFI, median fluorescent intensity. (*D*) The effects of the CB_1_ receptor antagonist AM251 on CBD-stimulated eNOS phosphorylation. Data are presented as mean ± SEM (*n* = 6) and were analysed by ANOVA with Sidak's multiple comparison test of selected pairs. **P < 0.01, ***P < 0.001.

To establish the intracellular mechanisms activated by CBD, human aortic endothelial cells were treated for 10 min with increasing concentrations of CBD. This led to a significant reduction in phosphorylated JNK (*Figure 5B*), NFκB (*Figure 5C*), p70s6 K (*Figure 5G*), and STAT5 (*Figure 5I*). CBD also significantly increased phosphorylated CREB (only at 30 μM, *Figure 5A*), ERK1/2 (*Figure 5E*), and Akt (*Figure 5F*). In the presence of the CB_1_ receptor antagonist AM251 (100 nM) or the TRPV1 antagonist capsazepine (1 μM), CBD no longer significantly increased phosphorylated ERK1/2 (*Figure 6A*). The increase in phosphorylated Akt was only inhibited by AM251 (*Figure 6B*). The levels of phosphorylated ERK1/2 (*P* = 0.0379, R = 0.3639) and Akt (*P* = 0.0343, R = 0.3749), but none of the other intracellular signalling pathways, were positively correlated with the increase in phosphorylated eNOS levels (*Figure 6C*). In the presence of AM251, the increase in phosphorylated eNOS was no longer significant (*Figure 6D*).

**Figure 7:**
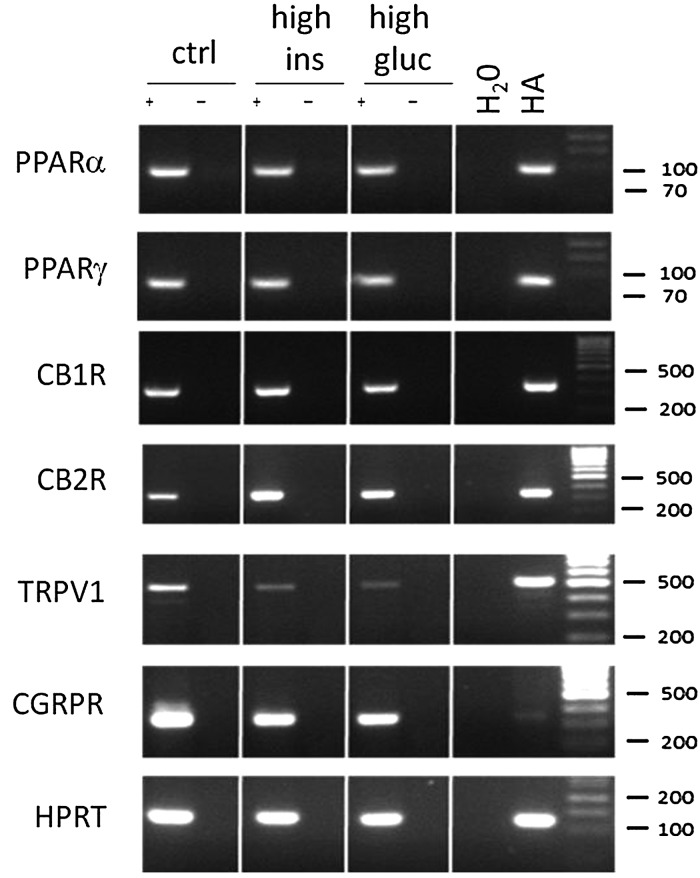
The effects of high insulin and glucose on the expression of cannabinoid targets in HAECs. RT-PCR showing the presence of PPARα and γ, CB_1_, CB_2_, TRPV1, CGRP receptors, and a house-keeping gene hypoxanthine-guanine phosphoribosyltransferase (HPRT) in human aortic endothelial cells (HAECs) grown in control conditions (first column) or a high insulin (500 nM, second column) or high glucose (25 mM, third column) environment for 96 h. Human astrocytes (HA) are shown as a positive control for cannabinoid targets.

As the CBD vasorelaxant responses were blunted in patients with type-2 diabetes, we carried out RT-PCR in human aortic endothelial cells (HAECs) to establish the effects of a high glucose (25 mM) or high insulin (500 nM) environment on the expression of the relevant target sites at the RNA level. Human astrocytes were used a positive control for these target sites.^23^ In HAECs, all targets (PPARα and γ, CB1R, CB2R, TRPV1, and CGRPR) were found to be present in control conditions (see *Figure 7*). After 96 h in either a high insulin or high glucose medium, the expression of CB2R appeared increased, and the expression of TRPV1 and CGRPR appeared decreased (see *Figure 7*).

## 4. Discussion

This is the first study to show that CBD-induces vasorelaxation in human mesenteric arteries which is dependent on CB_1_ and TRP receptor activation, the endothelium, nitric oxide, and potassium channel modulation. CBD-induced vasorelaxation is reduced in males, and in patients with type-2 diabetes, hypercholesterolemia and in patients taking statins, beta blockers and hypoglycaemic medication.

We found that CBD causes half-maximal vasorelaxation with a *p*EC_50_ in the mid-micromolar range. Similar findings have been reported in the rat mesenteric artery, where CBD causes vasorelaxation with mid-micromolar potency, however, in the rat model CBD caused near maximal vasorelaxation. This might suggest that the efficacy of CBD is reduced in human vasculature. However, it should be noted that the present studies were performed in older patients with a variety of comorbidities and medications, while animal studies are performed in same gender, young homogenous populations. As we observed that some diseases and medications were associated with lower responses to CBD, this might account for the apparent reduced efficacy in humans. As no mechanistic studies with CBD in animal tissue have yet been reported, we cannot compare the mechanisms of action established in the present study with that from animal tissue.

The endothelium mediates vasorelaxation of the CBD analogue Abn-CBD, and this vasorelaxation is associated with activation of the CB_e_ receptor which is antagonized using O-1918.^21,29^ We also found that removal of the endothelium reduced responses to CBD and that CBD vasorelaxant responses correlated with bradykinin responses, indicating an endothelial site of action for CBD. However, in the presence of O-1918, CBD-induced vasorelaxation is unaltered, suggesting that the endothelial component is not CB_e_. We also found that CBD responses tended to be reduced in the presence of l-NAME. To explore this further, we found that CBD significantly increased the phosphorylation of eNOS in human aortic endothelial cells, suggesting that production of NO a least partially underlies the endothelium-dependent vasorelaxant effect of CBD. The present study also reports that CBD-induced vasorelaxation is significantly inhibited in arteries contracted using high potassium solution, as has been shown for the vascular response to many cannabinoids. This suggests a predominant mechanism of CBD-induced vasorelaxation is activation of potassium channels and subsequent hyperpolarization. Given the extent of inhibition caused by KPSS, it is unlikely that potassium channel involvement is exclusive to the endothelium.

Activation of CB_1_ and CB_2_ receptor has been implicated in cannabinoid-induced vasorelaxation.^1^ Since human vascular smooth muscle and endothelial cells express these receptors,^30–35^ and CBD has been shown to bind to these receptors at low micromolar concentrations,^36,37^ they were considered as potential mechanisms underpinning CBD-induced vasorelaxation. Antagonism of the CB_1_ receptor in two separate experiments using AM251 (see *Figures 3 and 4*) revealed inhibition of CBD-induced vasorelaxation, suggesting CB_1_ is a target for CBD. A second structurally different antagonist, LY320135, was also found to inhibit the vasorelaxant response to CBD, further implicating CB_1_ receptor activation. Other authors have suggested that CBD may have indirect actions at CB_1_ through inhibition of FAAH activity or transport,^30^ rather than direct activation. However, we have previously shown that CBD is a more efficacious vasorelaxant of human mesenteric arteries that anandamide^38^ and that the mechanisms of action of CBD presented in the present study are different to those revealed recently in our laboratory for the endocannabinoid 2-AG.^39^ Despite this, CBD has low affinity for CB_1_ receptors so the possibility still exists that some of the actions of CBD are through inhibition of endocannabinoid degradation. Antagonism of the CB_2_ receptor using AM630 did not inhibit CBD-induced vasorelaxation. This was unsurprising as CB_2_ receptor activation is not commonly found to underpin the vasorelaxant effects of cannabinoids.^1^

The CB_1_ receptor is expressed in both human endothelial cells and vascular smooth muscle cells.^32,35^ In order to establish the location of the CB_1_ receptor mediated the vasorelaxant response to CBD, we compared responses with CBD in arteries both denuded and treated with AM251 to either intervention alone. Although the reduction in the maximal response to CBD was similar in arteries treated with AM251 alone as to both interventions, the entire response to CBD (represented by the AUC data) was more significantly reduced by the combination of both interventions. We take this data to suggest that CBD acts at CB_1_ located on both the endothelium and smooth muscle. CB_1_ activation has been shown to be coupled to the release of NO.^40^ In support of this, we found that in human endothelial cells, CBD increased the phosphorylation of eNOS, the mRNA of CB1R was present, and in the presence of AM251, the increase in eNOS phosphorylation by CBD was no longer significant.

Plant-derived cannabinoids are good activators of the TRPV channel family^41^ and CBD induces cancer cell apoptosis^42^ and anti-hyperalgaesic responses to inflammatory pain^43,44^ through activation of TRPV channels. In the present study, desensitization of TRP channels by exposure to the TRPV1 agonist capsaicin inhibited CBD-induced vasorelaxation, implicating TRP activation. In the rat mesenteric artery, vasorelaxation to two chemically closely related cannabinoids, THC and cannabinol, are also inhibited by capsaicin pre-treatment, acting via the release of the vasoactive neuropeptide calcitonin gene-related peptide (CGRP).^45^ Recent work showed that CGRP vasorelaxant responses in human arteries are endothelium-independent,^46^ suggesting the residual relaxation to CBD observed after endothelium-denudation is probably the TRP component of this response. However, we also observed that the increase in ERK caused by CBD in human endothelial cells was inhibited by TRPV1 antagonism, indicating that TRP activation on both the endothelium and smooth muscle cells could mediate some of the effects of CBD.

In the rat aortae, CBD causes time-dependent vasorelaxation that can be inhibited by PPARγ antagonism.^22^ In human small mesenteric arteries, we found that CBD-induced vasorelaxation also gradually increases with time, but this effect was not inhibited by PPARγ antagonism. However, we previously observed in rats that PPARγ mediated time-dependent vasorelaxant responses to cannabinoids were only observed in conduit arteries such as the superior mesenteric artery and aorta, but not in third-order mesenteric arteries.^47^ Thus the lack of PPARγ-mediated vasorelaxation seen to CBD may be due to the size of the arteries in the present study. An interesting observation was that the vasorelaxant response to CBD was non-recoverable, persisting up to 2 h post-administration. This is in contrast to our previous observations with THC^47^ where tone recovered. However, the mechanisms of action (CB_1_, NO, and the endothelium) of CBD reported in the present study are very different to that reported for THC.^48^

Human endothelial cell-based studies showed that CBD causes a range of intracellular signalling pathways to be altered at concentrations from 100 nM, but not in a classical concentration-dependent manner. This non-classical concentration–response, particularly for ERK and Akt activation, may be a result of activation of multiple targets by CBD. Indeed the ERK activation appeared to be inhibited by antagonists of both CB_1_ and TRPV1. Bell-shaped response curves to CBD are also commonly observed.^49,50^ The observed phosphorylation of ERK and Akt is consistent with known CB_1_-mediated signal transduction, and CB_1_-mediated activation of ERK has been observed in human umbilical vein endothelial cells.^35^ Indeed, we found that CB_1_ antagonism prevented this increase in ERK. Cannabinoid activation of both MAPK and Akt in the vasculature has also been suggested to be via non-CB_1_/CB_2_ mechanisms such as CB_e_.^51,52^ However, given our response to CBD was not antagonized by O-1918, it is unlikely that CBD acts through this site. Vasorelaxation to many compounds is mediated by activation of ERK and Akt, thus the CBD-induced increased in both ERK and Akt and therefore both may represent the intracellular signalling mechanisms underpinning the vasorelaxant effects of CBD, as suggested by the positive correlation with eNOS phosphorylation and the inhibition of eNOS phosphorylation by AM251.

CBD also significantly decreased the level of phosphorylated JNK and NFκB, key pro-inflammatory pathways, in human endothelial cells. This is consistent with previous studies showing CBD can attenuate the increase in JNK and NFκB caused by hepatic ischemia/reperfusion injury,^53^ diabetic cardiomyopathy,^11^ and hyperglycaemia.^12^ Our data suggest that reductions in these inflammatory pathways in endothelial cells may underpin some of the protective effects of CBD observed in the vasculature.^5^

Previous studies have shown a decrease in the phosphorylation of p70s6K, an mTOR substrate, in response to synthetic CB_1/2_ agonist^54^ or THC^55^ in cancer cells linked to autophagy pathways. STAT5 is also crucial in the regulation of cell fate, and its activation is key in angiogenesis.^56^ The reduction in the levels of phosphorylated p70s6K and STAT5 in human endothelial cells in response to CBD in the present study may represent the intracellular signalling mechanisms underpinning the anti-angiogenic effects of CBD reported by Solinas *et al.* ^57^ in human umbilical vein endothelial cells.

Given the variability of the responses seen to CBD, *post hoc* analysis of patient medical notes was undertaken. We found that CBD-induced vasorelaxation was enhanced in females compared with males. The enhanced vasorelaxation observed in female patient arteries compared with males may be due to protective effects of oestrogen on endothelial function.^58^ It has also been shown that CB_1_ receptor expression was increased in the leucocyte cells of females when compared with males.^59^ CBD responses were also reduced in those with increased cholesterol or diabetes. In rats fed high cholesterol diets, CB_1_ receptor expression is reduced.^60^ Similarly, CB_1_ expression is reduced (and associated vasorelaxant responses to anandamide) in obese rats.^60^ To test whether this might also be true in human aortic endothelial cells, we carried out RT-PCR on the major targets for cannabinoid in control condition and after prolonged exposure to a high-glucose or high-insulin environment. We did not find a reduction in CB1R expression; however, CB2R expression did appear to be up-regulated, which is in agreement with numerous studies showing that CB_2_ is up-regulated in vasculature pathologies.^61^ We did however also observe a decrease in the expression of TRPV1 and CGRPR in response to both a high-glucose or high-insulin medium, which is consistent with reports showing that TRPV1 vasorelaxant responses and receptor coupling to nitric oxide is disrupted in diabetes.^62^ Interestingly, the TRPV1 receptor has a cholesterol binding site which reduces its function.^63^ Thus the blunted CBD response in diabetic and hypercholesteraemia patients may be as a result of reduced TRPV1 expression and/or function, which warrants further investigation. Several medications (beta blockers, statins, and oral hypoglycaemics) were also associated with reduced vasorelaxant responses to CBD, but it is not yet clear whether this represents a drug–drug interaction, or whether it is a result of the pathology for which the medication is being taken.

In conclusion, this study reports that CBD causes vasorelaxation of the human mesenteric artery. This vasorelaxation is mediated through CB_1_, TRP channels, the endothelium and potassium channel activation. CBD also causes time-dependent vasorelaxation of human mesenteric arteries, but this was not due to PPARγ activation. The vasorelaxant effects of CBD are reduced in patients with hypercholesterolemia and type-2 diabetes, which may be a result of a reduced TRPV1 component. In human endothelial cells, CBD causes alterations in the phosphorylation of many intracellular proteins that might explain the vasorelaxant (eNOS, ERK, and Akt), anti-inflammatory (JNK and NFκB) and anti-angiogenic effects of CBD (p70s6K and STAT5).

## Supplementary material

Supplementary Material is available at *Cardiovascular Research* online.

## Funding

This work was supported by the British Heart Foundation (FS/09/061). Funding to pay the Open Access publication charges for this article was provided by the British Heart Foundation.
